# Which Role for Hyperbaric Oxygen Therapy in the Treatment of Fournier's Gangrene? A Retrospective Study

**DOI:** 10.3389/fsurg.2022.850378

**Published:** 2022-04-06

**Authors:** Roberta Tutino, Francesco Colli, Giovanna Rizzo, Sebastiano Bonventre, Gregorio Scerrino, Giuseppe Salamone, Giuseppina Melfa, Giuseppina Orlando, Gaetano Gallo, Mauro Santarelli, Marco Massani, Gianfranco Cocorullo

**Affiliations:** ^1^Chirurgia 3, Dipartimento di Chirurgia Generale e Specialistica, Azienda Ospedaliero Universitaria Città della Salute e della Scienza di Torino, Turin, Italy; ^2^Department of Surgical, Oncological and Stomatological Sciences, University of Palermo, Palermo, Italy; ^3^Department of General Surgery, University of Catanzaro, Catanzaro, Italy; ^4^Chirurgia 1, Ospedale Regionale di Treviso, Azienda ULSS 2 Marca Trevigiana, Treviso, Italy

**Keywords:** Fournier's gangrene, hyperbaric therapy, fasciitis, perineum, necrosis

## Abstract

**Purpose:**

In Fournier's gangrene, surgical debridement plus antimicrobial therapy is the mainstay of treatment but can cause a great loss of tissue. The disease needs long hospital stays and, despite all, has a high mortality rate. The aim of our study is to investigate if factors, such as hyperbaric therapy, can offer an improvement in prognosis.

**Methods:**

We retrospectively evaluated data on 23 consecutive patients admitted for Fournier's gangrene at the University Hospital “P. Giaccone” of Palermo from 2011 to 2018. Factors related to length of hospital stay and mortality were examined.

**Results:**

Mortality occurred in three patients (13.1%) and was correlated with the delay between admission and surgical operation [1.7 days (C.I. 0.9–3.5) in patients who survived vs. 6.8 days (C.I. 3.5–13.4) in patients who died (*p* = 0.001)]. Hospital stay was longer in patients treated with hyperbaric oxygen therapy [mean 11 (C.I. 0.50–21.89) vs. mean 25 (C.I. 18.02–31.97); *p* = 0.02] without an improvement in survival (*p* = 1.00).

**Conclusion:**

Our study proves that a delay in the treatment of patients with Fournier's gangrene has a correlation with the mortality rate, while the use of hyperbaric oxygen therapy seems to not improve the survival rate, increasing the hospital stay instead.

## Introduction

Fournier's gangrene (FG) comprises all necrotizing fasciitis of the perineum, regardless of the etiology, with or without proven infection. It has an incidence rate of ~1.6 per 100,000 males in western regions (1). The etiopathogenesis is debated between a primary ischemic process and infection because it is unclear if the disease represents an ischemic process complicated by infection from commensals or an infection finally causing the thrombosis of small subcutaneous vessels (2).

The related mortality ranges from 3 to 45%, with an overall rate of 16% proposed by a recent review (3). Deaths are due to severe sepsis, coagulopathy, acute renal failure, diabetic ketoacidosis and multiple organ failure.

The Fournier's gangrene severity index score (FGSIS) (Table 1) and the simplified FGSI score (SFGSI score) (Table 2), which includes only creatinine, hematocrit, and potassium are useful to stratify the disease (4, 5). In the low-risk group, according to the SFGSI, 1.3% of mortality has been reported, in contrast with 41% in the high-risk one (6).

**Table 1:** Fournier's Gangrene Severity Index (FGSI): >9 LRINEC = 75% probability of death; <9 LRINEC = 78% probability of survival.

**Variables**	**High abnormal values**				**Normal**	**Low abnormal values**			
Points	+4	+3	+2	+1	0	+1	+2	+3	+4
Temperature (C)	>41	39–40.9	–	38.5–35.9	36–38.4	34–35.9	32–33.9	30–31.9	<29.9
Heart rate	>180	140–179	110–139	–	70–109	–	56–69	40–54	<39
Respiration rate	>50	35–49	–	25–34	12–24	10–11	6–9	–	<5
Serum Na+ (mmol/l)	>180	160–179	155–159	150–154	130–149	–	120–129	111–119	<110
Serum K+ (mmol/l)	>7	6–6.9	–	5.5–5.9	3.5–5.4	3–3.4	2.5–2.9	–	<2.5
Serum Creatinine (mg/100 ml) (×2 for acute renal failure**)**	>3.5	2–3.4	1.5–1.9	-	0.6–1.4	–	<0.6	–	–
Hematocrit (%)	>60	-	50–59	46–49.4	30–45.9	–	20–29.9	-	<20
White blood count (total/mm^3^ x 1,000)	>40	–	20–39	15–19.9	3–14.9	–	1–2.9	–	<1
Serum bicarbonate (venous, mmol/l)	>52	41–51.9	–	32–40.9	22–31.9	–	18–21.9	15–17.9	<15

**Table 2:** Simplified Fournier's gangrene severity index (SFGSI): >2 = High risk patients; ≤ 2 = Low risk patients.

**Variables**	**High abnormal values**				**Normal**	**Low abnormal values**			
Points	+4	+3	+2	+1	0	+1	+2	+3	+4
Serum K+	>7	6–6.9	–	5.5–5.9	3.5–5.4	3–3.4	2.5–2.9	–	<2.5
Serum creatinine (x2 for acute renal failure)	>3.5	2–3.4	1.5–1.9	–	0.6–1.4	–	<0.6	–	–
% Hematocrit	>60	–	50–59	46–49.4	30–45.9	–	20–29.9	–	<20

*Low risk patients: 1.3% of mortality. High-risk patients: 41.0% of mortality [LIN 2]*.

Surgical debridement with broad spectrum antimicrobial therapy is the main treatment of FG (7). This broad- spectrum therapy is suggested regardless of the Gram stain and culture results, of course it can be reassessed when results are obtained (2).

In FG, the infection and edema reduce local blood circulation and tissue oxygenation, which increase the progression of necrosis, impair host defenses, and permit invasion of microorganisms. Tissue hypoxia is determined by two main factors: the reduction of blood flow in the amicted tissues and the concomitant proliferation of aerobic bacteria. Thus, this decrease in local oxygen concentration facilitates the seeding and spread of anaerobic bacteria, while causing thrombosis and tissue ischemia (8). Adequate debridement can cause a great loss of tissue, whose healing process can take a longer time, which is confirmed by the long hospital stays.

In this scenario, hyperbaric oxygen therapy (HBOT) could potentially be a therapeutic option to speed wound healing as it increases tissue oxygen tension to a level that inhibits and kills anaerobic bacteria, reduces systemic toxicity, limits the necrotizing fasciitis and enhances the demarcation of gangrene (9).

To investigate the role of HBOT in the treatment of FG, we retrospectively evaluated the patients admitted at the O.U. of General Surgery and Emergency of the University Hospital “P. Giaccone” of Palermo from January 2011 to November 2018.

## Materials and Methods

Data on 23 consecutive patients admitted at the Urgent and General Surgery O.U. of the University Hospital “P. Giaccone” of Palermo who underwent surgical operations for FG were retrospectively collected. The patients were identified on admission by the diagnostic code of the ICD-9: 608.83. For each patient, we collected demographic data, admission characteristics, and management and treatment results from the charts of patients. Demographic data collected included age, sex, body mass index (BMI), comorbidities, American Society of Anesthesiologists (ASA) score, and delay from symptoms to admission.

Admission characteristics included laboratory values, radiological findings, and microbiological stains. The SFGSI score was calculated for each patient. Data on perioperative management included time until first operation, number and type of operative procedures, need for colostomy, type of anesthesia, type of antibiotic therapy and HBOT. As outcomes, we ultimately recorded length of hospital stay (HS) and 30-day mortality.

### Statistical Analysis

We conducted this statistical analysis to examine the potential relationship between the use of HBOT, length of hospital stay and mortality.

Descriptive data are presented as parametric and non-parametric data.

The relation between the simplified FGSI score and the use of HBOT, the need for a diverting stoma (colostomy) and the length of hospital stay were evaluated using the Chi-square test or the independent-sample *t*-test when appropriate.

Possible factors influencing mortality and specifically sex, age, BMI, comorbidities, ASA score, duration of symptoms, simplified FGSI score, use of HBOT, need for colostomy and need for several operations were investigated using the independent-sample *t*-test, Welch test or Fisher's exact test.

Statistical analysis was conducted using MedCalc statistical software (MedCalc Software, Ostend, Belgium).

## Results

A total of 23 patients (16 M and seven F with mean age 62.7 years, sd 13.1, C.I. 37–84) were admitted between 2011 and 2018 for FG and underwent surgery.

The comorbidities of patients are showed in Table 3. The average BMI was 29.4 kg/m^2^ (sd. 6.5, C.I. 20.5–47.75).

**Table 3:** Patients' comorbidities.

**Patients' comorbidities**	
Tobacco consumption	50%
Alcohol abuse	15%
Diabetes	55%
COPD	13.1%
Cardiovascular diseases	34.8%
Inflammatory bowel disease	13.1%
Arthritis	8.7%
Cronic renal failure	13.1%
Cronic liver disease	8.7%
Cancer on chemotherapy	13%

The average duration of symptoms before admission was 11 days (sd 7.9, C.I. 3–30).

First location of symptoms was gluteal in five patients (21.7%), inguinal in four patients (17.4%), perineal in eight patients (34.8%) and scrotal in six patients (26.1%). The average white blood cell count at admission was 21,000 (sd 10,300), and average neutrophil count was 80.1% (sd. 17.9). C-reactive protein was > 1.25 mg/dl in 47% of patients. Fever was present in only three patients (18.8%) and bulging in seven patients (43.8%).

The diagnosis was supported by an ultrasound examination in eight patients (34.8%), and almost all patients received a CT evaluation. Air bubbles were found on CT in 69.5% of patients, fluid collections in 52.2% of patients, and soft tissue edema in 43.5% of patients.

The average delay between admission and surgery was 4 days (sd 4.4, C.I. 0.1–17); < 24 h in 30% of patients, 24–48 h in 22% of patients, 48–72 h in one patient and more than 72 h in 43.5% of patients.

The ASA score was I [0 patients], II (2), III (5), IV (8), V (1); six of the patients were managed only with local anesthesia.

Empiric broad-spectrum antibiotic therapy [penicillins (for gram positive), clindamycin or metronidazole (for anaerobes) and cephalosporine with aminoglycosides or fluoroquinolones (for gram negative) or, as alternative, monotherapy with carbapenems or piperacilline-tazobactam] was always administered and successively modified accordingly to the results of the samples (Table 4). The average length of antibiotic therapy was 22 days (sd 11, C.I. 10–60).

**Table 4:** Patient data.

	**Sex**	**Age**	**Site**	**Signs**	**Lin-score**	**ASA**	**Anesthesia**	**Colostomy**	**Antibiotics**	**Bacterial isolation**	**HBOT**	**LOS**	**Death**
1	M	68	Thigh root	fever and bulging	6	5	General	0	Daptomicina, metronidazol, Levofloxacin, meropenem	*Escherichia coli*	Not fit for	21	0
2	M	49	Perineum	Pain	11	3	Sedation	0	Clindamicin, imipenem cilastatin, daptomicin, vancomicin, meropenem, metronidazol	1	15	0	
3	F	61	Groin	Bulging and pain	10	4	General	0			0	44	0
4	M	72	Perineum	Fever	8	4	General	1	Metronidazol, daptomicin, meropenem		1	28	0
5	F	57	Thigh root	Fever	7	4	General	0	Clindamicin, pip-tazo, daptomicin	*Enterococcus fecalis*	1	20	1
6	F	69	Perineum	Pain and bulging	9	4	Sedation	0	Ceftazidim, metronidazol		1	47	0
7	F	76	Buttock	Fistulizing and bulging	12	4	General	1	Daptomicin, pip-tazo, metronidazol	*Stafilococco aureus, E. fecalis, E. coli*	1	21	1
8	M	84	Scrotum	Bulging	7	4	Sedation	0			1	37	0
9	M	72	Scrotum			-	Local	0			0	16	0
10	M	37	Scrotum/penis		10	-	Local	0			0	67	0
11	M	57	Scrotum	Pain and bulging	2	-	Local	0	Levofloxacin		0	7	0
12	F	55	Thigh root	Pain and bulging	9	4E	Sedation	0	Clindamicin, pip-tazo, Anidulafungin, linezolid	0	14	0	
13	M	57	Perineum		4	3	General	1			1	21	0
14	F	81	Buttock	Fistulizing	6	3	General	0	Teicoplanin, metronidazolo, Colimicin	*S. aureus*	1	31	0
15	M	64	Groin	Edema and erythema	1	2	General	0	Amoxicillin-clavulanat		1	13	0
16	M	42	Perineum			2	General	0			0	72	0
17	M	81	Perineum	Pain and edema	4	3	General	0	Clindamicin, ceftazidim, imipenem, vancomicin	*E. coli, Candida albicans*	1	27	0
18	M	71	Scrotum			-	Local	1			0	38	1
19	M	59	Perineum	Pain and bulging	4	-	Local	0	Cefixim, metronidazol		0	3	0
20	F	56	Buttock	Bulging	2	-	Local	0	Cefixim		0	5	0
21	M	73	Scrotum			4	General	0			0	15	0
22	M	63	Buttock	Pain	2	3	General	0	Linezolid, cefotaxim, clindamicin, ampicillin+sulbactam	*Streptococcus anginosus*	1	15	0
23	M	39	Perineum	Pain and bulging	6	2	Sedation	0	Clindamicin, daptomicin, tigeciclin, meropenem	*E. coli, Streptococccus sanguinis, Enterococcus fecalis*	0	20	0

*Demogrhapics: sex, age, signs, Lin's score, ASA. Intervention: type of anesthesia, need for colostomy. Admistered antibiotics. Bacterial isolation. Post-surgical HBOT administration. Length of hospital stay. Mortality*.

A colostomy was performed in four patients (17.4%). A total of 10 patients (43.5%) needed more than one surgical procedure. HBOT was offered to 13 patients (56.5%) using a scheduled session of 60 min daily.

An adverse event represented by dyspnea, sweating and agitation was reported during HBOT.

The average length of hospital stay was 26 days (sd. 17.9, C.I. 3–72). Mortality occurred in three patients (13.1%), two being treated with HBOT.

The length of hospital stay was influenced neither by the need for colostomy (*p* = 0.21) nor by SFGSI score > 2 (*p* = 0.68).

The use of HBOT did not improve the need for colostomy (*p* = 0.50) or several operations (*p* = 1.00).

HBOT use was not related to patients' severity of disease according to FSGI score (*p* = 1.00).

Hospital stay was longer in patients treated with HBOT [mean 11 (CI 0.50–21.89) vs. mean 25 (CI 18.02–31.97); *p* = 0.024].

Investigating factors related to mortality, the lapse between admission and surgical operation was the only statistically related to mortality, being 1.7 days (C.I. 0.9–3.5) in patients who survived vs. 6.8 days (C.I. 3.5–13.4) in patients who died (*p* = 0.001); other factors investigated, such as sex (*p* = 0.20), BMI (*p* = 0.53), renal failure (*p* = 1.00), diabetes (*p* = 0.49), age > 65 years old (*p* = 0.55), SFGSI score > 2 (*p* = 0.05), higher ASA score (≥ 4) (*p* = 0.47), symptoms lasting since more than 72 h before admission (*p* = 0.28), HBOT (*p* = 1.00), need for colostomy (*p* = 0.06), several operations (*p* = 1.00), and several operations plus HBOT (*p* = 1.00) did not show a relation.

## Discussion

There are different opinions and studies on the use of HBOT in this type of patient (Table 5) (7, 10–21). In a series of 11 patients, Pizzorno et al. attributed a 0% mortality rate to the adoption of HBOT (9).

**Table 5:** Literature reports on the use of HBOT in Fournier's gangrene.

**References**	**N. of patients**	**Days of hospital stay**		**Mortality**		
		**HBOT**	**Without HBOT**	**HBOT**	**Without HBOT**	**Total**
Pizzorno et al. (9)	11	NR	–	0	–	0
Korhonen et al. (7)	33	36	–	9.1%	–	9%
Mindrup et al. (10)	42	21	25	26.9% (7/26)	12.5% (2/16)	21.4%
Wagner et al. (11)	41	23	–	0	–	0
Janane et al. (12)	70	6	–	11.4%	–	11.4%
Martinschek et al. (13)	8	NR	–	12.5%	–	12.5%
Li et al. (14)	28	31	31	12.5% (2/16)	33.3% (4/12)	21.43%
Hung et al. (15)	60			0	66.7% (32/48)	32/60
Milanese et al. (16)	6	NR	–	0	–	0
Ferretti et al. (17)	20	22	34	0 (0/4)	18.75% (3/16)	15%
Ayan et al. (18)	41			0 (0/18)	39% (9/23)	
Hollabaugh et al. (19)				7%	42%	
Baraket et al. (20)	20	NR	NR	0 (0/4)	25% (4/16)	20%
Our study	23	25	11	15.4% (2/13)	10% (1/10)	13%

Accordingly, none of the patients who underwent HBOT died in the series proposed by Ayan et al. (18). Another positive outcome came from a study done by Mehl et al., where patients with FG who were given HBOT with routine surgical treatment had a mortality rate of 11.5%, whereas the mortality rate was 35.7% for those who underwent only conventional surgical treatment. Thus, the study concluded that patients who were treated with HBOT had a lower mortality rate compared to conventional therapy alone (21).

Interestingly, Hollanbaugh et al. observed that the use of HBOT was statistically significant in 26 cases of FG, where mortality rate was 7%, and the index increased five times in patients who did not receive HBOT (19).

In contrast, recently, Rosa and Guerreiro reported a mortality rate of 20.8% in a series of 34 patients treated with HBOT (22).

In a larger retrospective study, Mindrup et al. found no difference in length of hospital stay or mortality in relation to HBOT, and the authors cautioned against the routine use of HBOT based on the cost associated with the therapy, $600–$1,300 per treatment at their center (10).

Shupak et al. pointed out through their study that HBOT, when used as a complementary treatment for necrotizing fasciitis, does not offer the advantage of decreasing morbidity and mortality. The outcome in their study among patients treated with HBTO showed a mortality rate of 36% for the treated group, while the untreated group had a mortality rate of 25%; the average number of episodes of surgical debridement per patient was also lower in the untreated group when compared to that in the treated group (23).

Similar outcomes were reported by Tharakaram and Keckes who also observed a lower number of episodes of surgical debridement in the untreated group. On the contrary, in their study, the mortality rate was lower in the group treated with HBOT vs. the group not treated with HBOT [12.5% (2/16) and 33.3% (4/12), respectively] (24).

In a study proposed by Stanley, analyzing 636 patients, the mortality rate of patients was reported to be 10.1% and was related to older age, higher BMI and lower WBC and platelet counts in a multivariate analysis. No data on the use of HBOT were reported in their analysis (25).

Differing from the high mortality rate found by a recent study by Rosa and Guerreiro (22), as well as from the no difference in length of hospital stay with the use of HBOT stated by Mindrup et al. (10), our study showed an increase in length of hospital stay in patients treated with HBOT [mean 11 (CI 0.50–21.89) vs. mean 25 (CI 18.02–31.97); *p* = 0.02] and no advantages in terms of mortality as assessed in 15.4% of patients in the HBOT group and 10% of patients in the non-HBOT group (*p* > 0.05).

In our study, the factor that adversely affected the prognosis was a delay more than 72 h between the emergency admission and the surgical debridement. The causes of delay can be due to missed diagnoses, theater availability, surgeons availability or initial conservative treatment with antibiotics only.

The progression of the disease is described by Horta as a four-step process with a first phase of 24–48 h of non-specific symptoms associated with local hardening, edema and erythema; a second phase that is considered invasive and presents with local and regional inflammatory manifestations; a third necrotic phase with a rapid worsening of the general state evolving into septic shock in 50% of the cases; and a fourth phase of healing or spontaneous restoration (26). The rapidity of progression of the gangrenous area is considered to be 2–3 cm/h (27).

Our data are in accordance with the ones reported by Lin et al. who suggest that early surgical interventions allow to maximize the survival benefit. Although the Authors recommend even shorter interval of times since they found that in high-risk patients (SFGSI score >2) mortality rate was 26.32% within 12 h, 40% between 12 and 24 h and 69.23% >24 h; early surgical interventions performed within 14.35 h from hospital arrival allowing to maximize the survival benefit (6).

So, when approaching patients, we have to remember that tenderness, erythema and swelling can mimic less severe infections, such as cellulitis and erysipelas; however, pain out of proportion to clinical examination should alert the clinician to the strong possibility of necrotizing fasciitis (28).

Our results are supported from the data reported by Yeniyol et al. in a study on 25 patients, where the authors report that mortality was related to both the FGSI and the difference in the duration of symptoms before admission, being 1.9 +/– 0.7 days in patients who survived and 4.1 +/– 1.4 days in patients who died (29).

Altarac reported that the median duration of symptoms before admission was a day longer in patients who not survived (4 days compared to 3 days), but this was not statistically associated with higher mortality (*p* = 0.11) (30).

Similar data have been reported by Basoglu et al.; in their study, the duration of the symptoms prior to gangrene in the survivors was 6.2 days (range 2–20 days) in comparison to 7.5 days (range 5–10 days) for the non-survivors (*p* > 0.05) (31).

Ersay et al. state that the median duration of symptoms at presentation was 7.00 days in survivors, but it was 8 days in non-survivors. The time from the onset of symptoms to presentation was not significantly different in survivors and non-survivors (*p* > 0.05) (32).

However, with the delay between symptoms and admission not being carefully predictable, our study focused the problem of prompt surgical treatment when patients are admitted. Thus, in our series, mortality was related to the delay of in-hospital treatment rather than on the delay between symptoms and admission. Of course, both delays are important for the cure rate, but only one of them being related in our series.

These data should underline the concept of the urgent situation when approaching a case of suspected FG and should encourage aggressive treatment each time the suspicion arises in a patient urgently admitted to the surgical department.

The current study has several limitations. It was retrospective and the number of patients was quite small, but this can be explained by the rarity of the disease. The gravity of the disease was evaluated with the SFGSI score instead of the FGSI because not all the data to calculate this score were present.

## Conclusions

In our study, patients treated with HBOT showed an increase in length of hospital stay, and HBOT did not offer an improvement in mortality when added to surgical debridement plus antibiotic therapy.

As previously suggested, the incoming necrosis has to be promptly stopped when the suspicion of FG first arises, because the delay in treatment seems to be the most important factor causing an increase in mortality and the only factor in our study that worsened the prognosis of patients.

## Data Availability Statement

The raw data supporting the conclusions of this article will be made available by the authors, without undue reservation.

## Ethics Statement

Ethical review and approval was not required for the study on human participants in accordance with the local legislation and institutional requirements. The patients/participants provided their written informed consent to participate in this study.

## Author Contributions

RT and FC have given substantial contributions to the conception or the design of the manuscript. GR, GSc, GSa, and GM have given substantial contributions to the acquisition, analysis and interpretation of the data. RT, FC, and GR have participated to drafting the manuscript. GG, SB, MS, MM, and GC revised it critically. All authors read and approved the final version of the manuscript.

## Conflict of Interest

The authors declare that the research was conducted in the absence of any commercial or financial relationships that could be construed as a potential conflict of interest.

## Publisher's Note

All claims expressed in this article are solely those of the authors and do not necessarily represent those of their affiliated organizations, or those of the publisher, the editors and the reviewers. Any product that may be evaluated in this article, or claim that may be made by its manufacturer, is not guaranteed or endorsed by the publisher.

## Abbreviations

FG: Fournier's gangrene
FGSI: Fournier's gangrene severity index score
SFGSI score: Simplified Fournier's gangrene severity index score
HBOT: hyperbaric oxygen therapy
BMI: body mass index
ASA: American Society of Anesthesiologists score
HS: Hospital stay.
